# Chronic and Acute Low Back Pain After Spinal Anesthesia: A Systematic Review and Meta-analysis

**DOI:** 10.5812/aapm-169715

**Published:** 2026-02-28

**Authors:** Alireza Shakeri, Firoozeh Madadi, Soudeh Tabashi, Seyed Mohammad Seyed Alshohadaei, Alireza Jolous Jamshidi, Ardeshir Tajbakhsh

**Affiliations:** 1 Anesthesiology Research Center, Shahid Beheshti University of Medical Science, Tehran, Iran; 2 School of Medicine, Shahid Beheshti University of Medical Sciences, Tehran, Iran; 3 Clinical Research Development Center, Imam Hossein Educational Hospital, Shahid Beheshti University of Medical Sciences, Tehran, Iran

**Keywords:** Spinal Anesthesia, Back Pain, Acute Pain, Chronic Pain, Postoperative Complications, Systematic Review And Meta-analysis

## Abstract

**Context:**

Spinal anesthesia is commonly used for many surgical procedures because of its rapid onset and reliable anesthetic effects. Nevertheless, postoperative lower back pain (LBP) remains a frequent complication, with reported rates varying substantially across the literature. This systematic review and meta-analysis aimed to synthesize the available evidence and estimate the overall incidence of both short-term acute and long-term persistent LBP following spinal anesthesia.

**Evidence Acquisition:**

Major databases, including PubMed, Scopus, Embase, and Web of Science, were systematically searched to identify studies of LBP after spinal anesthesia. Following data extraction, relevant outcomes were pooled for analysis. Given the expected methodological heterogeneity across studies, a random-effects model was used for data synthesis. Between-study heterogeneity was quantified using Cochran's Q test and the I^2^ statistic. Publication bias was assessed using funnel-plot inspection and established statistical methods.

**Results:**

The review included 15 studies comprising 4,478 patients, of which 11 provided sufficient data for meta-analysis. The pooled overall rate of postprocedural LBP was 25.65% (95% CI: 19.54% - 32.29%), with substantial between-study heterogeneity (I^2^ = 96.84%). Subgroup analyses showed an acute LBP prevalence of 21.96% (95% CI: 16.01% - 28.56%) and a chronic LBP prevalence of 44.98% (95% CI: 26.95% - 63.73%). Marked heterogeneity persisted across these subgroups. Most included studies involved orthopedic, urologic, or obstetric/gynecologic surgery.

Discussion: Back pain is frequently reported after spinal blocks during both short- and long-term recovery phases. However, substantial methodological heterogeneity among the primary studies precludes the establishment of a definitive causal relationship. Well-designed prospective studies using standardized assessment tools are needed to clarify the clinical implications of these findings.

## 1. Introduction

Among patients undergoing surgery with spinal anesthesia, lower back pain (LBP) is a commonly reported adverse event (1, 2). Although this postprocedural discomfort is generally benign and self-limiting, a subset of patients experience prolonged pain that substantially impairs daily functioning, occupational duties, and physical mobility (3, 4). Such complications are particularly common in obstetric and orthopedic practice, in which prolonged operative postures, mechanical stress on the spine, and procedural factors can further increase spinal loading (5, 6).

Current epidemiological data on this adverse event are inconsistent. The literature reports widely divergent incidence rates, largely because of heterogeneous study designs, diverse patient populations, variable observation periods, and the lack of universal definitions of acute versus chronic pain (7). In addition, primary trials rarely adjust for key confounders in a standardized manner, including pre-existing spinal conditions, body mass index (BMI), spinal needle gauge, and the number of needle passes (8). These methodological disparities hinder robust interstudy comparisons and obscure the true clinical burden of LBP after neuraxial blockade.

Given the fragmented nature of the evidence, a comprehensive reevaluation of the literature is warranted. Clarifying the epidemiological distinction between transient postoperative backache and persistent spinal pain may improve postoperative surveillance and preoperative counseling. Accordingly, this meta-analysis was designed to calculate the pooled prevalence of both acute and chronic LBP after spinal anesthesia based on the most current evidence.

## 2. Methods

### 2.1. Study Protocol and Registration

This systematic review was conducted in accordance with the updated PRISMA 2020 statement (9). To ensure methodological transparency and minimize the risk of reporting bias, the study protocol was prospectively registered in the PROSPERO international database (Identifier: CRD420251183454). Further details regarding data extraction and outcome handling are provided in the supplementary documents.

### 2.2. Search Strategy and Study Selection

A comprehensive literature search was conducted across four major electronic databases: PubMed/MEDLINE, Embase, Scopus, and Web of Science. Articles published up to January 16, 2024, were included. The primary search was supplemented by manual screening of the bibliographies of relevant articles and by a Google Scholar search. The search strategy combined specific keywords and standardized vocabulary, including MeSH terms, related to "spinal anesthesia" and "low back pain" (the detailed search syntax is provided in Table S1 in Supplementary File).

All retrieved citations were exported to EndNote X9 for deduplication. Two investigators independently conducted a blinded screening process, beginning with title and abstract screening and followed by full-text assessment against predefined eligibility criteria. Disagreements between the two investigators were resolved through consensus discussion or arbitration by a third senior author. Of the 37 articles assessed at the full-text stage, 15 studies met the inclusion criteria for quantitative synthesis, as shown in the PRISMA flow chart (Figure 1).

**Figure 1.**
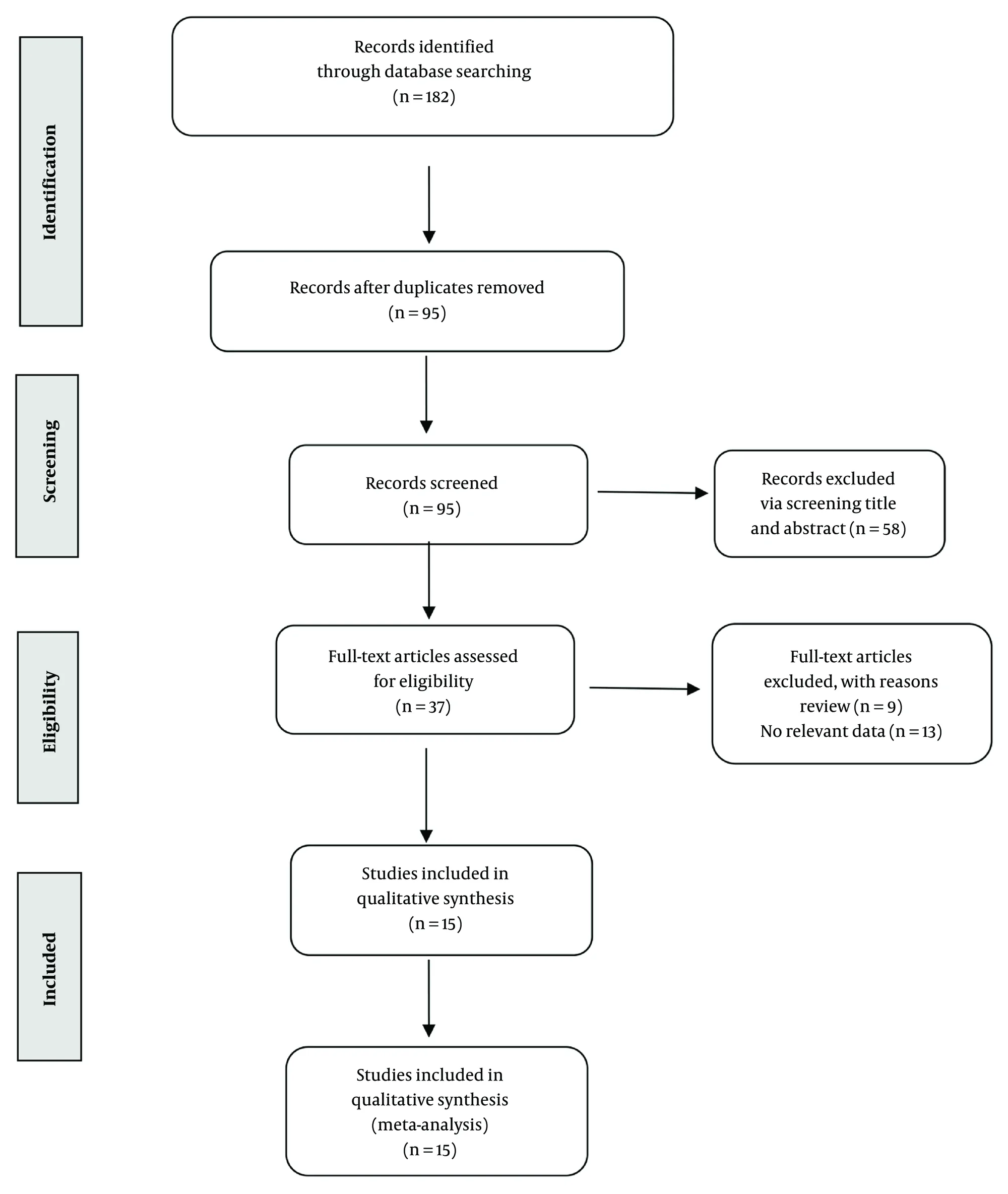
PRISMA flow diagram illustrating the selection of articles

### 2.3. Data Extraction, Terminology, and Risk-of-Bias Assessment

To ensure accuracy and minimize bias, two researchers independently extracted all essential data using a predefined standardized template. Extracted information included the first author, country, study design, sample size, surgical category, anesthesia-related details, and the reported frequencies or criteria for backache after the intervention.

For analytical uniformity, the timing of pain onset was preclassified. Symptoms occurring within the first 4 postoperative weeks were categorized as acute LBP, whereas persistent pain lasting at least 3 months was categorized as chronic LBP. Studies that did not report precise follow-up timelines were omitted from the quantitative synthesis but retained for narrative synthesis.

The methodological quality and risk of bias of the included studies were assessed using design-specific tools. Randomized controlled trials were evaluated using the Cochrane Risk of Bias 2 (RoB 2) tool, cohort studies were assessed using the Newcastle-Ottawa Scale (NOS), and cross-sectional studies were evaluated using the Agency for Healthcare Research and Quality (AHRQ) checklist (10). Discrepancies in quality grading were resolved by an independent third reviewer. The complete quality-appraisal results are presented in Table 1.

**Table 1.** Risk-of-Bias Assessment of Included Studies ^a^

Studies	Study Design	Tool Used	Domain Scores	Overall Judgment
**Schwabe et al. (18)**	Prospective cohort	NOS	Selection: 4; Comparability: 1; Outcome: 2	7/9 (Good)
**Jin et al. (20)**	Prospective cohort	NOS	Selection: 4; Comparability: 1; Outcome: 2	7/9 (Good)
**Tekgül et al. (21)**	Cohort	NOS	Selection: 4; Comparability: 1; Outcome: 2	7/9 (Good)
**Forozeshfard et al. (12)**	Cohort	NOS	Selection: 3; Comparability: 1; Outcome: 2	6/9 (Fair)
**Peker et al. (17)**	Cohort	NOS	Selection: 3; Comparability: 1; Outcome: 2	6/9 (Fair)
**Nathan et al. (16)**	Cohort	NOS	Selection: 3; Comparability: 1; Outcome: 2	6/9 (Fair)
**Mahdian et al. (22)**	Cross-sectional	AHRQ	Score: 7/11	Moderate risk
**Yirgu et al. (24)**	Cross-sectional	AHRQ	Score: 6/11	Moderate risk
**Tariq et al. (23)**	Cross-sectional	AHRQ	Score: 5/11	Moderate risk
**Zeleke et al. (25)**	Cross-sectional	AHRQ	Score: 8/11	Low risk
**Eidy et al. (11)**	Randomized controlled trial	RoB 2	Randomization: Low; Deviations: Low; Missing data: Low; Outcome measurement: Low; Reporting: Low	Low risk
**Khajavi et al. (14)**	Randomized controlled trial	RoB 2	Randomization: Some concerns; Deviations: Low; Missing data: Low; Outcome measurement: Low; Reporting: Low	Some concerns
**Singh et al. (19)**	Randomized controlled trial	RoB 2	Randomization: Low; Deviations: Low; Missing data: Low; Outcome measurement: Low; Reporting: Low	Low risk
**Lee et al. (15)**	Randomized controlled trial	RoB 2	Randomization: Low; Deviations: Low; Missing data: Low; Outcome measurement: Low; Reporting: Low	Low risk
**Jowkar et al. (13)**	Randomized controlled trial	RoB 2	Randomization: Low; Deviations: Low; Missing data: Low; Outcome measurement: Low; Reporting: Low	Low risk

^a^ Risk of bias was assessed according to study design. Randomized controlled trials were evaluated using the Cochrane Risk of Bias 2 tool. Cohort studies were assessed using the Newcastle-Ottawa Scale, and cross-sectional studies were evaluated using the Agency for Healthcare Research and Quality checklist. NOS scores were interpreted as follows: 7 - 9 = good quality, 4 - 6 = fair quality, and 0 - 3 = poor quality.

### 2.4. Statistical Analysis

Given the anticipated clinical and methodological diversity among the primary sources, a random-effects framework was used to estimate pooled prevalence proportions and corresponding 95% confidence intervals. Statistical inconsistency across studies was assessed using Cochran's Q test and the I^2^ statistic, with higher I^2^ percentages indicating substantial heterogeneity. Forest plots were generated to visually display the pooled outcomes (Table 2).

**Table 2.** Records Retained After Preliminary Filtering ^a^

Databases / Sources	Records Identified
**PubMed/MEDLINE**	68
**Scopus**	45
**Web of Science**	32
**Embase**	30
**Google Scholar**	5 ^b^
**Manual search**	2
**Total records identified before duplicate removal**	182
**Duplicate records removed**	87
**Records screened after duplicate removal**	95

^a^ The numbers presented in Table 2 reflect records retained after preliminary relevance filtering within each database, such as removal of clearly irrelevant clusters or technical duplicates, whereasTable S1 in Supplementary File presents the total raw records initially retrieved from each source before any filtering.

^b^ For Google Scholar, the first 200 results sorted by relevance were screened, of which 5 eligible studies were retained for further assessment.

Publication bias was assessed by visual inspection of funnel-plot asymmetry and by formal testing using Egger's regression. However, these findings were interpreted cautiously because of the limited number of included trials. When data permitted, a priori subgroup analyses were conducted according to surgical discipline and symptom duration. Sensitivity analyses were also performed by systematically excluding studies deemed to have a high risk of bias to assess the stability of the pooled estimates. Because the primary papers inconsistently reported key procedural factors, including needle gauge, operator expertise, and puncture frequency, meta-regression was not feasible. Review Manager (RevMan), version 5.4, was used for all quantitative analyses.

## 3. Results

### 3.1. Study Selection and Baseline Characteristics

As shown in the PRISMA flow chart (Figure 1), the exhaustive search strategy identified 15 eligible publications comprising a total of 4,478 patients. The quantitative synthesis included only 11 longitudinal investigations, including cohort studies and randomized controlled trials, that provided precise follow-up intervals required for temporal data aggregation (11-21). The remaining 4 cross-sectional studies did not report explicit symptom timelines and were therefore included only in the narrative synthesis (22-25).

The selected studies were published between 2001 and 2023 and covered diverse operative domains, predominantly gynecologic/obstetric, orthopedic, and urologic procedures. Mean participant ages varied widely, ranging from 28.5 to 65.45 years. The main demographic and methodological characteristics of the included studies are summarized in Table 3.

**Table 3.** Characteristics of Included Studies ^a^

First Author, Reference	Country	Study Design	Sample Size	Surgery Type(s)	Reported LBP (%)	Follow-up Period	Key Findings	Outcome Category
**Schwabe (18)**	Germany	Prospective cohort	245	General and trauma surgery	18.9 (prevalence)	3 months and 1 year	Pre-existing back pain was the only independent predictor of persistent postoperative back pain.	Overall; Chronic
**Jin (20)**	Singapore	Prospective cohort	857	Elective cesarean section	36.9 (prevalence)	Median 14.5 months	Higher postoperative pain scores and pain elsewhere were associated with chronic pain.	Overall; Chronic
**Eidy (11)**	Iran	RCT	176	Urologic surgery	18.2 (prevalence)	6 - 48 hours	Lidocaine and puncture at L3 - L4 were associated with higher LBP prevalence.	Overall; Acute
**Tekgül (21)**	Turkey	Prospective cohort	649	Orthopedic, urologic, general, cardiovascular	29.3 (prevalence)	1 day and 4 weeks	Bone contacts, prior back pain, larger needle size, and longer surgery duration increased LBP risk.	Overall; Acute
**Khajavi (14)**	Iran	RCT	220	General and urologic surgery	18.0 (prevalence)	3 - 90 days	No significant difference was observed between the midline and paramedian approaches.	Overall; Acute and Chronic ^b^
**Singh (19)**	India	RCT	50	Lower abdominal surgery	10.0 (prevalence)	≤ 7 days	The paramedian approach reduced postoperative headache and LBP.	Overall; Acute
**Mahdian (22)**	Iran	Cross-sectional	460	Gynecologic and mixed surgeries	47.6 (prevalence)	Not reported	Female sex, gynecologic surgery, and lateral position were associated with higher LBP prevalence.	Overall ^c^
**Yirgu (24)**	Ethiopia	Cross-sectional	318	Mixed surgical procedures	38.0 (prevalence)	1 day to 4 weeks	Previous back pain and spinal anesthesia technique were associated with LBP.	Overall; Acute
**Forozeshfard (12)**	Iran	Prospective cohort	410	General, orthopedic, urologic	5.8 (prevalence)	Up to 12 months	No significant association was observed between demographic or procedural variables and LBP.	Overall; Acute and Chronic
**Lee (15)**	South Korea	RCT	50	Mixed surgical procedures	36.0 (prevalence)	1 day to 3 months	The paramedian approach reduced early postoperative LBP.	Overall; Acute ^d^
**Tariq (23)**	Pakistan	Cross-sectional	100	Cesarean section	78.0 (prevalence)	≥ 3 months	A greater number of cesarean deliveries was associated with more severe LBP.	Overall; Chronic
**Peker (17)**	Turkey	Prospective cohort	362	Knee arthroscopy and arthroplasty	7.8 (prevalence)	Up to 1 week	Anxiety and depression scores correlated with postoperative LBP severity.	Overall; Acute
**Zeleke (25)**	Ethiopia	Cross-sectional	215	Mixed surgical procedures	40.5 (prevalence)	≤ 3 days	Higher BMI, larger needle size, multiple attempts, and bone contacts increased LBP risk.	Overall; Acute
**Jowkar (13)**	Iran	RCT	116	Lower limb orthopedic surgery	26.4 (prevalence)	1 day	No significant association was observed between demographic factors and postoperative LBP.	Overall; Acute
**Nathan (16)**	India	Retrospective cohort	250	Cesarean section	48.7 (prevalence)	> 6 months	No significant association was observed between spinal anesthesia and chronic LBP.	Overall; Chronic ^e^

^a^ Abbreviations: AHRQ, Agency for Healthcare Research and Quality; BMI, body mass index; LBP, low back pain; NOS, Newcastle-Ottawa Scale; PDPB, postdural puncture back pain; RCT, randomized controlled trial; RoB 2, Risk of Bias 2.

^b^ Khajavi et al. (14) included assessments extending to approximately 3 months; therefore, chronic outcomes were interpreted cautiously.

^c^ Mahdian et al. (22) was a cross-sectional prevalence study and was included only in the narrative synthesis and not in time-specific pooled analyses.

^d^ Lee et al. (15) reported follow-up assessments up to 3 months; however, outcomes beyond 4 weeks were not clearly stratified. Therefore, only acute postoperative LBP data were included in the pooled analyses.

^e^ No significant relationship was observed between spinal anesthesia-related variables and chronic LBP.

### 3.2. Methodological Quality and Risk of Bias

The overall quality of the included articles was assessed using tools appropriate for each study design and was generally acceptable. RoB 2 assessments showed that most included randomized controlled trials had a low risk of bias. Similarly, the cohort studies assessed using the NOS demonstrated fair-to-good quality, with scores ranging from 6 to 7 out of 9. For cross-sectional studies assessed using the AHRQ checklist, quality was generally consistent with a moderate risk of bias, except for 1 study judged to have a low risk of bias. Table 1 provides a detailed summary of these quality assessments.

In contrast to the initial version of this review, all included studies were reclassified according to their actual design (randomized, cohort, or cross-sectional), and design-specific tools were applied accordingly, as summarized in Table 1.

### 3.3. Meta-Analysis of LBP Prevalence

Pronounced statistical heterogeneity characterized all primary pooled estimates, with I^2^ values consistently exceeding 95%. Therefore, a random-effects analytical approach was used for all data synthesis.

The pooled prevalence of backache after spinal anesthesia was 25.65% (95% CI: 19.54% - 32.29%), with marked intertrial heterogeneity (I^2^ = 96.84%; Figure 2). When only acute manifestations were evaluated, the pooled incidence remained notable at 21.96% (95% CI: 16.01% - 28.56%), with similarly high heterogeneity (I^2^ = 96.12%; Figure 3).

**Figure 2.**
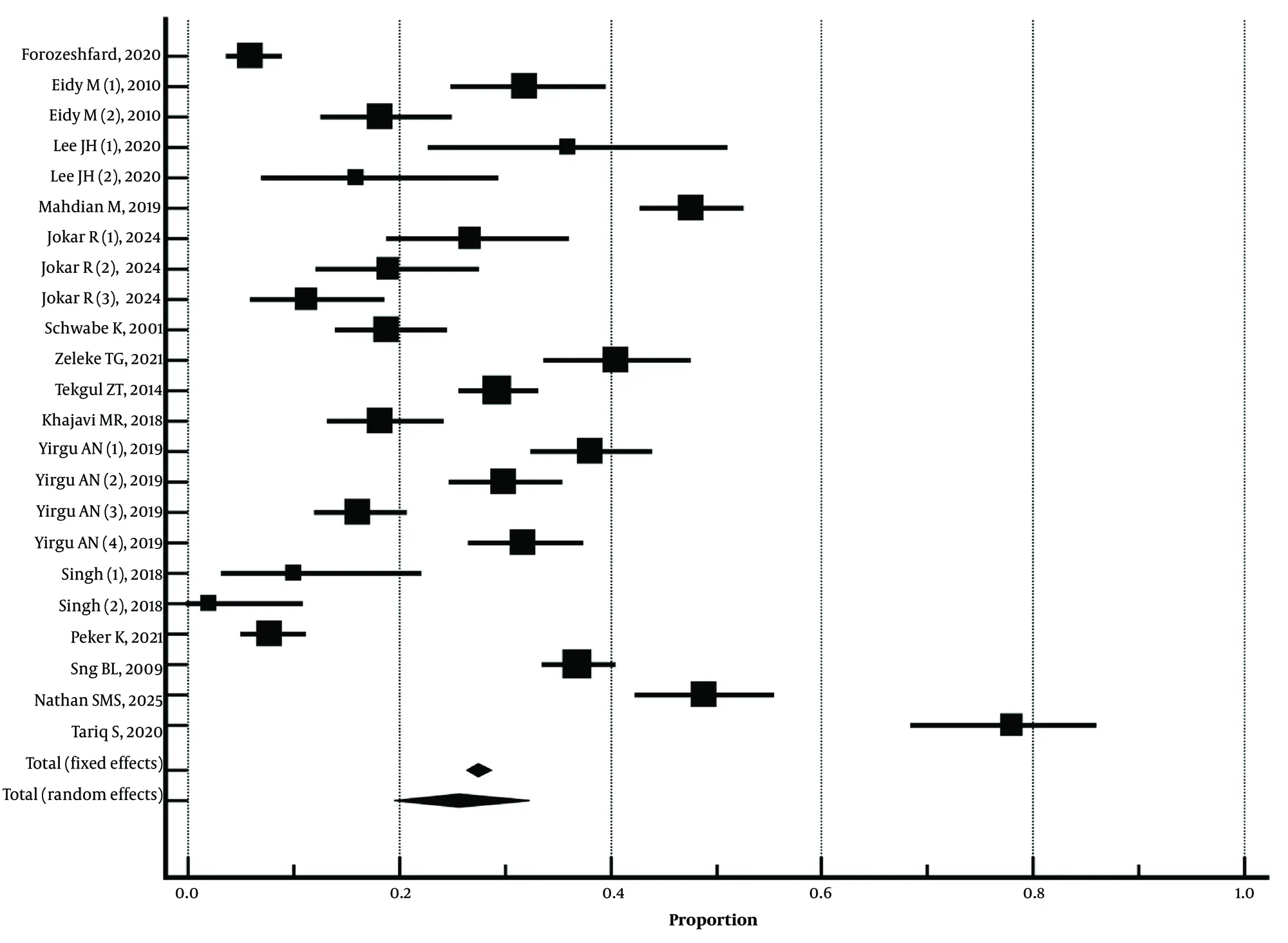
Forest plot of the meta-analysis of the prevalence of back pain following spinal anesthesia. The squares represent the effect size of each study with 95% confidence intervals, and the diamond represents the pooled effect size (11-25).

**Figure 3.**
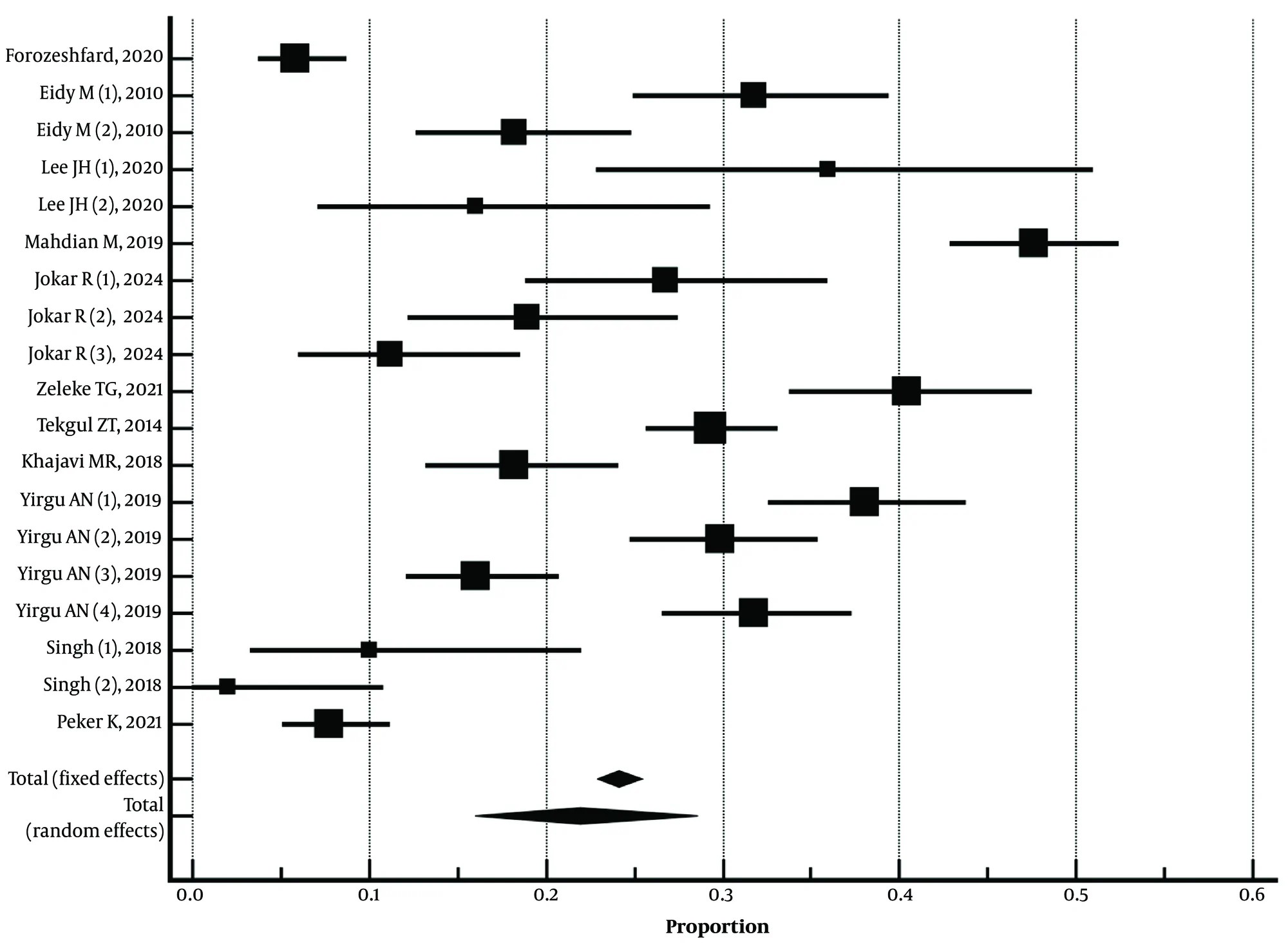
Forest plot of the meta-analysis of the prevalence of acute back pain following spinal anesthesia. The squares represent the effect size of each study with 95% confidence intervals, and the diamond represents the pooled effect size (11-15, 17, 19, 21, 22, 24, 25)

For studies evaluating long-term outcomes, the pooled rate of chronic LBP was 44.98% (95% CI: 26.95% - 63.73%). This subgroup showed the highest statistical heterogeneity observed in the current analysis (I^2^ = 97.64%; Figure 4).

**Figure 4.**
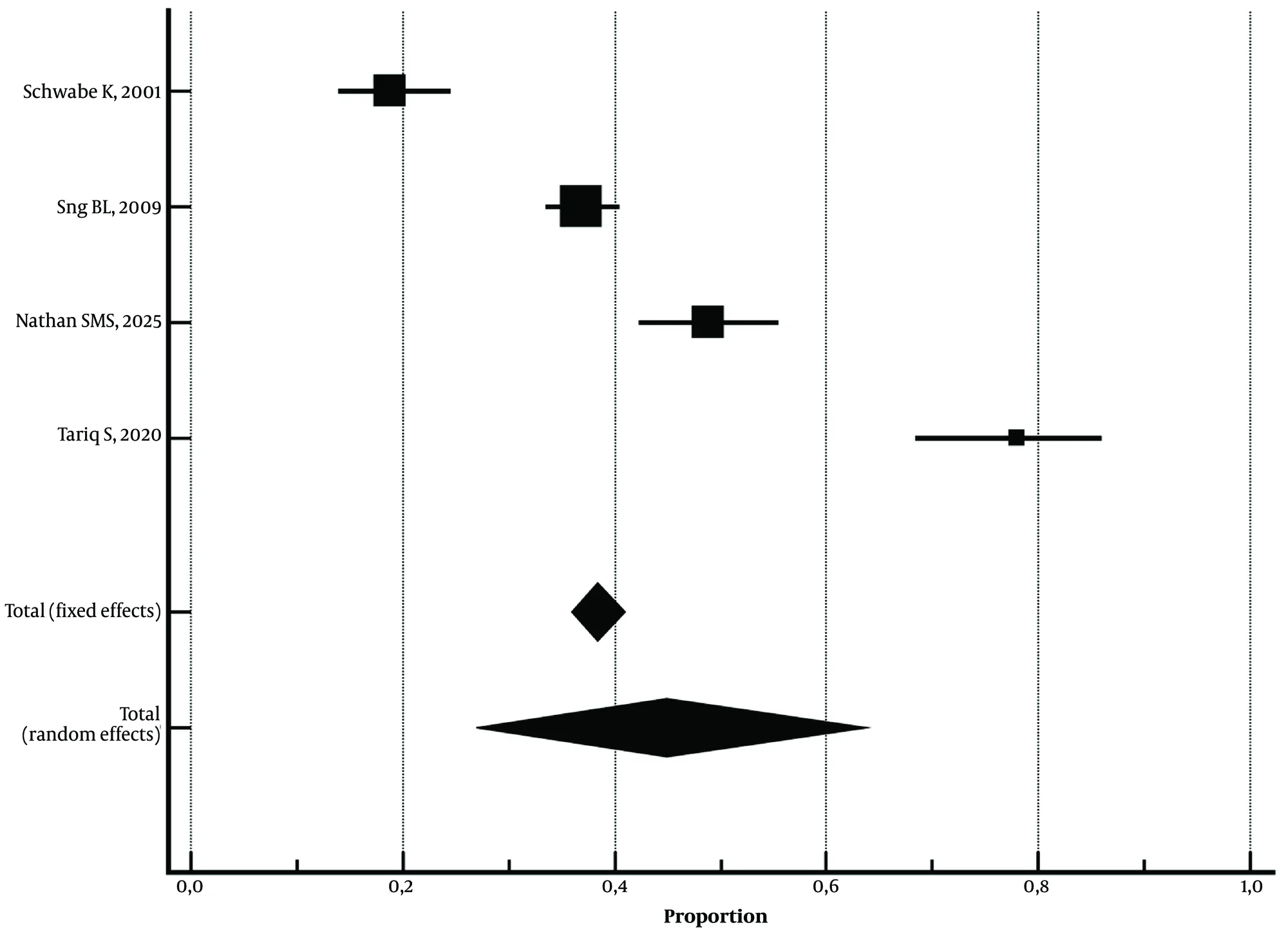
Forest plot of the meta-analysis of the prevalence of chronic back pain following spinal anesthesia. The squares represent the effect size of each study with 95% confidence intervals, and the diamond represents the pooled effect size 16, 18, 20, 23

### 3.4. Subgroup Analyses and Sensitivity Testing

To explore potential sources of pronounced statistical heterogeneity, subgroup analyses were performed according to surgical type by comparing obstetric cohorts with nonobstetric groups. Although this stratification produced slight variations in estimated prevalence, substantial dispersion persisted, with I^2^ values consistently exceeding 75%. Sensitivity analyses were also performed by systematically excluding studies judged to have an elevated risk of bias. These analyses produced only trivial changes in the primary pooled estimates, supporting the robustness of the central conclusions and indicating that the findings were not unduly influenced by methodologically weaker data.

### 3.5. Assessment of Publication Bias

Visual inspection of funnel-plot symmetry (Figure 5), together with formal quantitative assessment using Egger's and Begg's tests, indicated no overt publication bias or small-study effect (Table 4). However, these findings should be interpreted cautiously. The limited number of analyzed trials and the severe underlying heterogeneity reduce the validity of publication-bias detection methods, particularly when subgroup analyses include fewer than 10 studies.

**Table 4.** Assessment of Publication Bias ^a^

Outcomes	Egger's Test (P-Value)	Begg's Test (P-Value)
**Overall postoperative LBP**	0.483	0.567
**Acute postoperative LBP**	0.405	0.255
**Chronic postoperative LBP**	0.552	0.497

^a^ Egger's regression test and Begg's rank correlation test were used to assess potential publication bias. A P value < 0.05 was considered suggestive of statistically significant publication bias. Funnel plots were also visually inspected for asymmetry. However, because several subgroup analyses included fewer than 10 studies, the results of publication-bias tests should be interpreted with caution because of limited statistical power.

**Figure 5.**
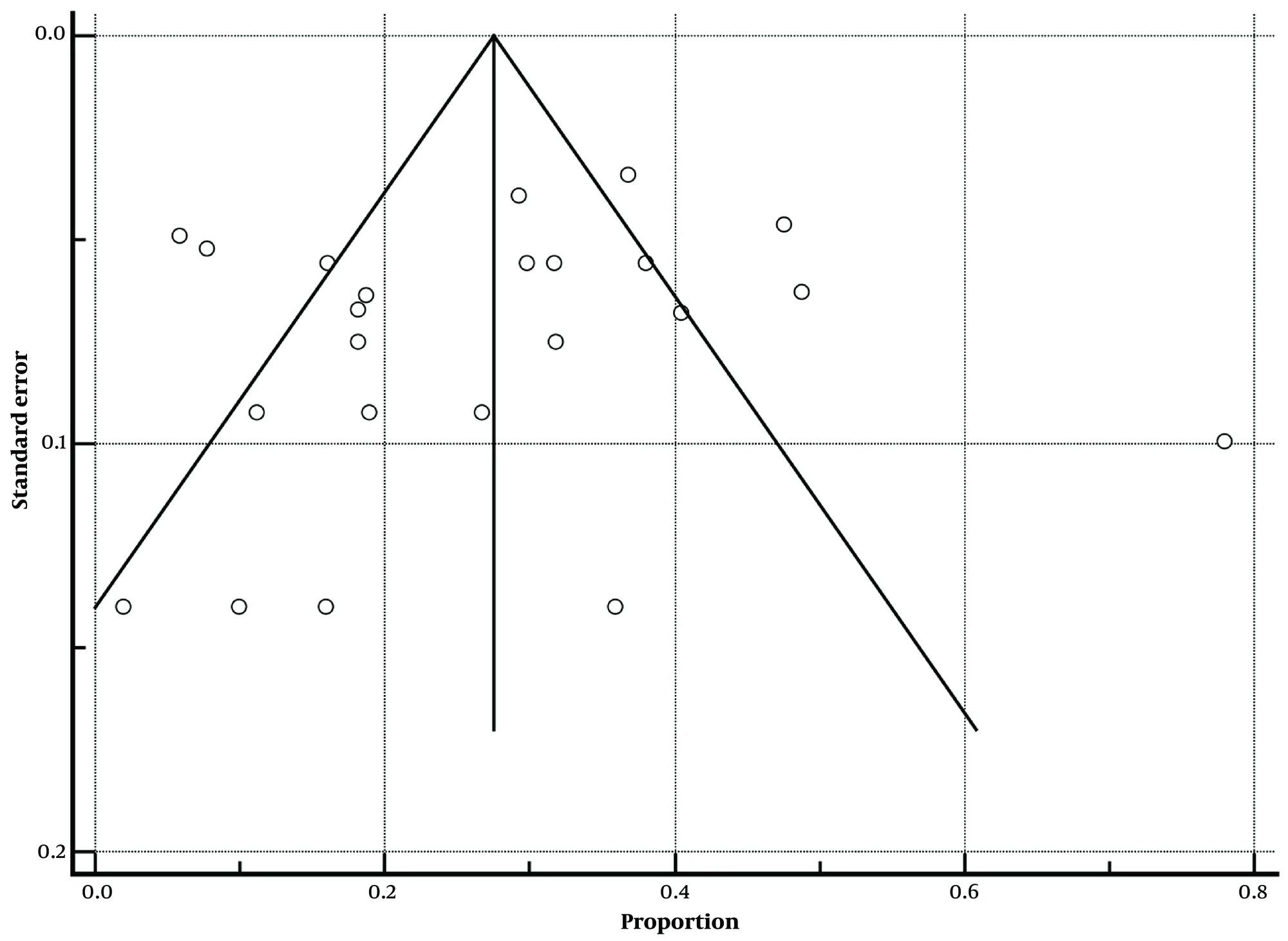
Funnel plot for assessing publication bias in studies reporting the overall prevalence of back pain.

## 4. Discussion

### 4.1. Principal Findings and Interpretation

This systematic review and meta-analysis was designed to determine the rate of LBP after spinal anesthesia across various surgical settings. The synthesized data indicated a considerable occurrence of these postoperative symptoms, with an overall pooled prevalence of 25.65%. When stratified by symptom duration, the acute LBP rate was 21.96%, whereas the chronic or prolonged backache rate was 44.98%.

However, these long-term prevalence estimates should be interpreted with considerable caution. The marked statistical dispersion underlying the pooled estimates indicates that variable diagnostic criteria, divergent follow-up durations, and heterogeneous patient profiles strongly influence the reported incidence rates. Therefore, the pooled estimates should not be regarded as absolute epidemiological values but rather as indicators of a wide range of possible postoperative outcomes. Overall, the synthesized evidence suggests that backache after spinal anesthesia remains a common complaint and is likely related to a complex interaction among patient-specific vulnerabilities, surgical tissue trauma, and mechanical factors associated with neuraxial puncture.

### 4.2. Alignment With Existing Literature

The quantitative findings are consistent with previous research indicating that lumbar discomfort after neuraxial anesthesia is frequent but multifactorial. A broad consensus in the literature suggests that persistent back pain arises from multiple contributing factors and cannot be attributed solely to the spinal injection.

In obstetric contexts, prior studies have found limited evidence supporting a direct causal association between spinal or epidural procedures and chronic postpartum back pain (16, 20, 23). Instead, such pain is often attributed to the physiological strain of childbirth, pregnancy-related biomechanical changes, and pre-existing spinal vulnerabilities. This interpretation is consistent with the substantial variance observed in the present analysis. Some studies reported persistent pain (18, 23), whereas others documented markedly low incidence rates (12, 17). These discrepancies are likely attributable to the inclusion of diverse surgical fields, such as urology, obstetrics, and orthopedics, within a single analysis, as well as inconsistent control of confounding variables and varied pain-assessment protocols (21, 22, 24, 25). Whereas earlier descriptive reviews focused primarily on theoretical mechanisms, the present study provides quantitative benchmarks for both acute and chronic phases.

Consistent with these findings, a recent meta-analysis by Timerga et al. (26) also reported insufficient evidence to support a definitive causal relationship between neuraxial anesthesia and chronic postpartum back pain, emphasizing instead the role of pregnancy-related biomechanical changes and pre-existing spinal conditions.

### 4.3. Underlying Mechanisms and Clinical Importance

The exceptionally high statistical heterogeneity, as reflected by the I^2^ values, likely results from a complex interaction between methodological inconsistency and true clinical variation. From a procedural perspective, factors such as needle gauge, multiple puncture attempts, traumatic ligament injury, and constrained patient positioning during the procedure have long been implicated in the development of LBP (11, 13, 14, 19).

Emerging evidence cautiously suggests that technical refinements, such as ultrasound guidance or a paramedian insertion technique, may reduce tissue trauma and improve targeting precision (15, 27). In addition, the choice of intrathecal pharmacological agents may influence the intensity of transient neurological side effects (28). However, the present meta-analysis was not designed to isolate the individual effects of these specific technical factors.

Beyond the anesthetic procedure itself, several nonanesthetic factors substantially affect postoperative comfort. Pre-existing degenerative spinal conditions, stressful intraoperative positioning, prolonged immobility, and inadequate postoperative rehabilitation may all contribute to postoperative LBP. Clinically, these findings emphasize the importance of clear preoperative counseling, meticulous needle-placement technique, and active management of modifiable risk factors before, during, and after the procedure, particularly because no definitive preventive strategy has yet been established.

### 4.4. Strengths and Limitations

Several strengths support the value of this systematic review. First, it provides a comprehensive synthesis of more than 2 decades of international research on this topic. Second, methodological rigor was enhanced through adherence to a preregistered PROSPERO protocol. Third, the use of clear chronological distinctions between acute and chronic pain improved consistency within the pooled dataset.

Several limitations should also be acknowledged. The most important limitation is the severe statistical heterogeneity (I^2^ > 90%) across the analyses, which inevitably reduces the precision of the pooled estimates, even after subgroup analyses. In addition, the available literature lacks standardization, with inconsistent follow-up schedules and variable pain-assessment methods. Key confounding variables, including procedure duration, BMI, history of lumbar pathology, and the number of unsuccessful puncture attempts, were reported too inconsistently to permit robust adjusted analyses.

Furthermore, the predominance of observational study designs introduces inherent risks of selection bias and residual confounding. The statistical power of publication-bias tools, such as Egger's test, is also limited when applied to a small number of studies. Finally, many included primary studies did not explicitly exclude patients with pre-existing musculoskeletal disorders. Therefore, some of the postoperative LBP identified in this analysis may represent exacerbation of pre-existing spinal conditions rather than truly de novo complications.

### 4.5. Conclusions

Low back pain after spinal anesthesia is frequently reported across diverse surgical settings. Although the pooled analysis suggests a substantial occurrence of both acute and persistent symptoms, the extensive statistical heterogeneity and methodological differences among the primary studies make it difficult to establish a definitive overall incidence rate. Importantly, the current body of evidence is inadequate to confirm a direct and independent causal link between neuraxial procedures and the development of chronic lumbar pain.

Instead, postsurgical backache should be viewed as a complex and multifactorial condition. Its etiology appears to be influenced more by the combination of pre-existing patient vulnerabilities, ergonomic factors related to surgical positioning, and general perioperative influences than by the anesthetic injection alone. These conclusions highlight the importance of thorough preoperative clinical evaluation, meticulous procedural technique, and vigilant postoperative monitoring. Future research should prioritize well-designed prospective studies that use standardized definitions of LBP, standardized follow-up intervals, and rigorous statistical control for known clinical confounders.

aapm-16-1-169715-s001.pdf

## Data Availability

The dataset presented in the study is available on request from the corresponding author during submission or after publication.
