# Supplementary material for: Chronic and Acute Low Back Pain After Spinal Anesthesia: A Systematic Review and Meta-analysis
Source: Anesth Pain Med. 2026 Feb 28;16(1):e169715. doi: 10.5812/aapm-169715 (PMC13768999; doi:10.5812/aapm-169715)
Supplement: Supplementary file 1 [file aapm-16-1-169715-s001.pdf]

Table S1. Raw Retrieval Counts

**Database**	**Search Date**	**Search Strategy**	**Records Retrieved**
PubMed/MEDLINE	15 January 2024	(“Spinal Anesthesia” [Mesh] OR “spinal anesthesia” OR “subarachnoid block”) AND (“Low Back Pain” [Mesh] OR “low back pain” OR lumbago OR backache)	68
Scopus	15 January 2024	TITLE-ABS-KEY (“spinal anesthesia” OR “subarachnoid block”) AND TITLE-ABS-KEY (“low back pain” OR lumbago OR backache)	45
Web of Science Core Collection	15 January 2024	TS= (“spinal anesthesia” OR “subarachnoid block”) AND TS= (“low back pain” OR lumbago OR backache)	32
Embase	16 January 2024	(‘spinal anesthesia’/exp OR ‘spinal anesthesia’ OR ‘subarachnoid block’) AND (‘low back pain’/exp OR ‘low back pain’ OR lumbago)	41
Google Scholar*	16 January 2024	Keywords: “spinal anesthesia” AND “low back pain”	18
Manual Reference Screening	16 January 2024	Screening of reference lists of eligible articles and relevant reviews	7

*Only the first 200 Google Scholar results sorted by relevance were screened, consistent with commonly recommended systematic review methodology.
